# Genetic analysis indicates spatial-dependent patterns of sex-biased dispersal in Eurasian lynx in Finland

**DOI:** 10.1371/journal.pone.0246833

**Published:** 2021-02-19

**Authors:** Annika Herrero, Cornelya F. C. Klütsch, Katja Holmala, Simo N. Maduna, Alexander Kopatz, Hans Geir Eiken, Snorre B. Hagen

**Affiliations:** 1 Natural Resources Institute Finland (Luke), Helsinki, Finland; 2 University of Helsinki, Helsinki, Finland; 3 NIBIO—Division of Environment and Natural Resources, Norwegian Institute of Bioeconomy Research, Svanvik, Norway; 4 Norwegian Institute for Nature Research (NINA), Trondheim, Norway; University of Nevada, Reno, UNITED STATES

## Abstract

Conservation and management of large carnivores requires knowledge of female and male dispersal. Such information is crucial to evaluate the population’s status and thus management actions. This knowledge is challenging to obtain, often incomplete and contradictory at times. The size of the target population and the methods applied can bias the results. Also, population history and biological or environmental influences can affect dispersal on different scales within a study area. We have genotyped Eurasian lynx (180 males and 102 females, collected 2003–2017) continuously distributed in southern Finland (~23,000 km^2^) using 21 short tandem repeats (STR) loci and compared statistical genetic tests to infer local and sex-specific dispersal patterns within and across genetic clusters as well as geographic regions. We tested for sex-specific substructure with individual-based Bayesian assignment tests and spatial autocorrelation analyses. Differences between the sexes in genetic differentiation, relatedness, inbreeding, and diversity were analysed using population-based AMOVA, F-statistics, and assignment indices. Our results showed two different genetic clusters that were spatially structured for females but admixed for males. Similarly, spatial autocorrelation and relatedness was significantly higher in females than males. However, we found weaker sex-specific patterns for the Eurasian lynx when the data were separated in three geographical regions than when divided in the two genetic clusters. Overall, our results suggest male-biased dispersal and female philopatry for the Eurasian lynx in Southern Finland. The female genetic structuring increased from west to east within our study area. In addition, detection of male-biased dispersal was dependent on analytical methods utilized, on whether subtle underlying genetic structuring was considered or not, and the choice of population delineation. Conclusively, we suggest using multiple genetic approaches to study sex-biased dispersal in a continuously distributed species in which population delineation is difficult.

## Introduction

Individual dispersal and gene flow into a breeding area impacts the evolutionary potential, long-term viability, and distribution ranges of populations and species [1–5]. Therefore, detailed understanding of species-specific dispersal patterns is required to forecast population responses to environmental change [2, 3]. Dispersal of terrestrial mammals is often sex-biased to avoid potential breeding of closely related individuals [6, 7]. Avoidance of inbreeding increases an individual’s fitness, but also affects the social and spatial genetic composition of a population [2, 4, 6–8]. By increasing the number of potential mates, successful immigrants may introduce new alleles and thereby increase genetic variation and effective population size [4, 6, 9].

Dispersal varies with several factors, including population size, landscape features, various anthropogenic disturbances, and especially by social systems of the species [10, 11]. Large terrestrial carnivores are often reported to display sex-biased dispersal with females showing lower dispersal rates and distances (i.e., sex-specific philopatry) compared to males [7, 12–14]. This is also true for the cat family *Felidae* [15], partly owing to their polygynous mating system [8]. Sex-bias in dispersal may, along with inbreeding avoidance, be driven by differing resource requirements, such as finding denning places for females and access to mates for males [8]. In the *Lynx* genus, sex-biased dispersal has been reported for bobcats (*Lynx rufus*) [14, 16, 17], but not for Canada lynx (*Lynx canadensis*) or Iberian lynx (*Lynx pardinus*) [18–20] and is less clear for Eurasian lynx. This study focuses on improving our understanding of dispersal dynamics in the Eurasian lynx.

Previously, telemetry studies of Eurasian lynx have documented dispersal of both sexes but cannot consistently ascertain that dispersal is sex-biased. In a Scandinavian telemetry study, males dispersed further and more frequently than females, of which one third remained philopatric [21]. In a Polish telemetry study, the few males that were studied predominantly dispersed further than the females [22]. In two Swiss telemetry studies, dispersal was similar for both sexes, but differed between areas of origin with sex as an additional factor [23, 24]. Furthermore, in a Finnish telemetry study, dispersal distances averaged about 38 km for females and 66 km for males, but both sexes showed large variation and dispersing equally short and long distances [25].

Genetic studies can detect the amount and direction of sex-biased gene flow as an indicator of sex-biased dispersal [12]. Eurasian lynx males and females inhabit their respective home ranges, the size of which depends upon the prey availability and landscape, and the degree of overlap on sex, lynx density and relatedness [26–28]. In a genetic study of Eurasian lynx in Poland, local females were more closely related with each other than males with other males [29]. Furthermore, in a small, isolated population in the western Carpathians, the genetic analysis showed that females were predominantly philopatric and reproduction was dominated by a few males, leading to inbreeding and sub-structuring into two family lineages [30]. Previously, we have detected strong evidence of local family genetic structuring in female Eurasian lynx in Finland, where about 64% of the females had a close relative (mother, daughter or sister) within an average of 37 km distance [11], but it remained unclear whether this suggested philopatry as only females were investigated [11]. In a large-scale study including several lynx populations, similar patterns of decreasing relatedness with increasing geographical distance, based on a spatial autocorrelation analysis, was observed for both sexes [31].

There may be several reasons for the observed variation across studies. The Eurasian lynx populations in Europe differ in size and level of connectedness with other populations [32], and, in fact many populations are small, isolated and fragmented [33] given historical bottlenecks and human-dominated landscapes [34]. Additionally, the different methodologies applied, and statistical approaches taken may influence the detection of sex-biased dispersal (e.g., telemetry *versus* genetic, see [3, 35–39], as well as the temporal and geographical scales studied [40]. Sex-biased dispersal can be studied using statistical genetic methods based on different principles that broadly fall into two categories. First, population-based statistical methods rely on pre-defined groups, using F-statistics [37, 41], analyses of molecular variance [42], and assignment indices [36, 37]. Second, individual-based statistical methods, which do not require pre-defined populations, such as spatial autocorrelation analysis [39] and Bayesian assignment methods like STRUCTURE [43]. The latter also allows immigrant and admixed individuals to be relatively easily identified [12, 44].

For population-based statistical methods, the required *a priori* delineation of groups to be analyzed may result in genetic structure and F-statistics estimates that may reflect historical rather than contemporary gene flow [43, 45]. Further, overall weak population-genetic structure, like in species that display high dispersal capabilities in both sexes, may make an *a priori* delineation of populations difficult [43, 46]. Two strategies have been applied to solve this [43, 46]. First, groups can be based on geographical regions as proxies or second, Bayesian clustering methods can be used to detect and delineate groups for further statistical analysis [43, 46, 47]. To our knowledge, the impacts of the different delineation methods on sex-biased dispersal tests are understudied, and none of the population-based statistical method will allow for analysis of within-population genetic patterns without introducing more artificial groupings [45, 46].

Individual-based statistical methods allow for the analysis of sex-biased dispersal by investigating the relationship between genetic and geographic distance for each of the sexes and require geographical coordinates [39]. These methods also require *a priori* groupings when local conditions are to be analyzed.

We have shown in a previous study on Eurasian lynx that underlying geographical structure can change the strength of the signal in spatial autocorrelation analyses [11]. Therefore, detection of sex-biased dispersal may require a correction factor for population genetic structure for species, in which generally subtle differences between the sexes are expected (e.g., both sexes have the ability for long-term dispersal). For population-based methods, it is predicted that under sex-biased dispersal, individuals of the dispersing sex show lower relatedness values [35], and F_ST_-values [36]. In the dispersing sex, a mix of resident and immigrant individuals are expected to be present, leading to larger heterozygosity deficit and consequently, higher F_IS_-values than in the philopatric sex [36].

In addition, the dispersing sex should display generally weaker structure in Bayesian clustering methods than the philopatric sex because the former will homogenize the gene pool across the landscape [35, 36]. For spatial autocorrelation analyses, our predictions include that the dispersing sex should show weak or no spatial pattern of relatedness between individuals whereas for the philopatric sex, a pattern of spatial genetic relatedness should be visible [31, 39, 46]. Finally, assignment indices calculate the probability of an individual’s multilocus genotype originating in the population in which it was collected [35–37]. The dispersing sex should therefore display negative assignment indices while the philopatric sex displays positive indices.

In this study, we have used both population-based and individual-based statistical analyses to infer sex-specific dispersal patterns from genetic data in a continuously distributed population of Eurasian lynx in southern Finland. Eurasian lynx inhabit all of Finland, and forms a continuously distributed population in connection with that of Russian Karelia, [48] but remain separate from the Scandinavian population [31]. While nearly eradicated from Finland by the early 20^th^ century, the population received natural recruitment from Russian Karelia [49]. Lynx recolonization in the wild started in the 1960’s from east to west with the most prominent range expansion and population growth occurring during late 1990s and early 21st century [49]. Yearly derogation hunting of Finnish lynx is regulated by the Ministry of Forestry and Agriculture and has varied from about 10–20% of the estimated total population size during the study period [50]. The hunting is non-selective, although females with kittens of the year are principally protected. The yearly hunting bag of lynx in Finland typically includes approximately 60/40% males/females [51].

We applied 21 bi-parentally inherited short tandem repeat (STR) markers to individuals sampled after dispersal (> 1 year), to obtain unbiased estimates [12] of sex-biased dispersal [35]. Kittens are born in May-June and juveniles of both sexes typically disperse between April and July the following year, on average at the age of ten months old [21, 23, 25]. Natal dispersal is the most common with adult dispersal rare. By building upon the results of our previous study on female Eurasian lynx [11], that documented matrilineal assemblages in the study area, we tested the null hypothesis of equal dispersal by the sexes by estimating the degree of sex-specific patterns in dispersal, spatial organization, and genetic diversity indices. Our prediction was that males will disperse farther than females on average [11], and thus show lower genetic differentiation and relatedness than females. Alternatively, both sexes would show similar dispersal and structuring patterns. We also investigated how population- and individual-based genetic tests of sex-biased dispersal differ in their power and bias depending on the groups selected for analysis, i.e. genetic clusters or pre-defined geographical regions [12, 45].

## Materials and methods

### Study area, sampling, and age estimation

**Fig 1:**
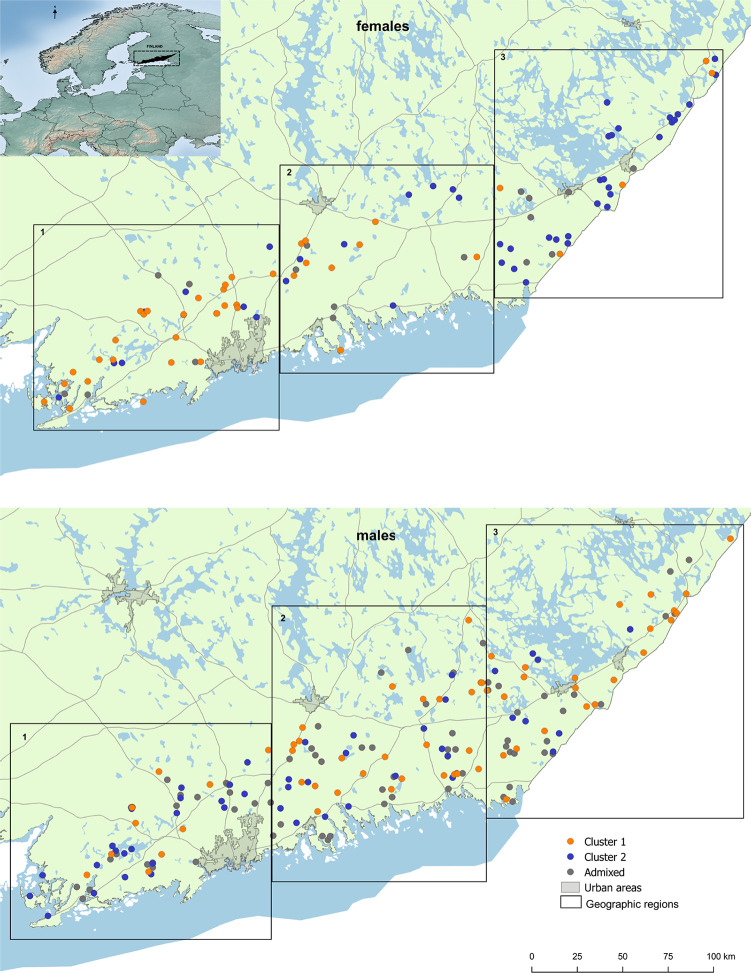
The study area for Eurasian lynx in Southern Finland. Spatial distribution of the two genetic clusters for a) female and b) male Eurasian lynx (N_male_ = 180; N_female_ = 102) as well as admixed individuals in grey. Black boxes indicate the three geographical regions used for the LOCPRIOR function in STRUCTURE and as an alternative grouping for summary statistics studied. Reprinted from National Land Survey of Finland 06/2020 under a CC BY 4.0 license, with permission from CSC—IT Center for Science Ltd, urn:nbn:fi:att:64cdb2e7-0b10-48f2-bfea-ed5c089d8de3, original copyright 2019.

Our study area is in southern Finland (WGS 84: Lat 59°– 61°, Lon 22°– 29°, Fig 1), covering a total area of 22,936 km^2^. Forest dominated by pine, spruce, and birch constitutes about 70% of the terrestrial area. Lakes encompass about 10% and the rest consists of mires, farmland, and urban areas, creating a blend of different land uses. Despite the high forest cover, the area holds five of the 15 largest urban areas in Finland, including the capital area of Helsinki, which lies about 100 km east from the western-most point of the study area. Human population density varies between 6 to 3,044 individuals / km^2^. Our initial data set consisted of all 369 legally hunted lynx during 2003–2017 (238 males and 131 females). Tissue samples for genetic analysis were collected from all individuals at the Natural Research Institute Finland’s (Luke) lab in Taivalkoski Research Station. No ethic permit was required, as the samples did not involve live animals. A CITES permit was obtained for shipping the samples to the Norwegian Institute of Bioeconomy Research (NIBIO) lab in Svanvik, Norway. The tissue samples were stored in ethanol until analysis. The age of the individuals was determined from cementum annuli analysis of tooth samples in a laboratory specialized in age determination (Matson´s Laboratory, Montana, USA) [52]. Before the analyses of sex-biased dispersal, 0-year olds were excluded to avoid the analysis for sex-bias to be affected by undispersed individuals [35]. In the study area, the hunting season is from the beginning of December to the end of February. Thus, the location of a shot lynx aged one year or over, most likely represents an established post-dispersal home range in a local population. This resulted in a final dataset (n = 282) consisting of 180 males and 102 females (Table 1 and S1 Table in S1 Material).

**Table 1:** Genetic diversity estimates for two genetic clusters and three geographical groups of Eurasian Lynx in southern Finland during 2003–2017.

Group	N	H_O_ (SE)	H_E_ (SE)	F_IS_	P value
**CL1F**	29	0.632 (0.046)	0.602 (0.037)	-0.033	0.906
**CL1M**	46	0.627 (0.045)	0.615 (0.037)	0.009	0.678
**CL2F**	52	0.651 (0.034)	0.654 (0.030)	0.014	0.219
**CL2M**	50	0.671 (0.022)	0.660 (0.024)	-0.006	0.635
**ADXF**	21	0.653 (0.040)	0.634 (0.032)	-0.006	0.595
**ADXM**	84	0.658 (0.032)	0.663 (0.029)	0.014	0.160
**WESTF**	40	0.630 (0.039)	0.613 (0.033)	-0.016	0.776
**WESTM**	54	0.673 (0.032)	0.658 (0.028)	-0.012	0.769
**CENTRALF**	22	0.641 (0.041)	0.647 (0.031)	0.033	0.125
**CENTRALM**	72	0.647 (0.028)	0.658 (0.027)	0.023	0.056
**EASTF**	40	0.664 (0.036)	0.659 (0.033)	-0,004	0.447
**EASTM**	54	0.643 (0.032)	0.662 (0.032)	0.038	**0.019**

The analyzed groups are named according to genetic clusters (CL1, CL2) and the admixed groups (based on q threshold of <70%): admixed females (ADXF) and admixed males (ADXM). Geographical regions are named from west to east (WEST, CENTRAL, and EAST). N = number of individuals. H_O_ (SE) = observed heterozygosity with standard error, H_E_ (SE) = expected heterozygosity with standard error, F_IS_ = inbreeding coefficients, F = females, M = males. P values = P value for inbreeding coefficient F_IS_ (adjusted P = 0.0004 based on 2520 randomizations, [38]) using the software FSTAT. Significant results at the 0.05 level in bold.

### DNA extraction and genotyping

Procedures for DNA extraction and genotyping are outlined in detail in [11]. Briefly, after DNA extraction from tissue samples with the DNeasy 96 Blood and Tissue kit (Qiagen), samples were genotyped at 21 short-tandem repeat loci (STRs) [53, 54] in seven multiplexes [11]. Compared to [11], the use of two STR markers (Fca078 and Fca001) was discontinued in the present study due to time-consuming allele scoring. Molecular sex determination was done by amplifying regions on the zinc finger region on the felid X- and Y-chromosome with primers developed by [55]. Amplification reactions contained 5.0 μl 2x Multiplex PCR MasterMix (Qiagen), 1.0 μl Primer mix (see Table 1 in [11] for concentrations of each primer set), 0.05 μl BSA (NEB), and 2.95 μl ddH2O in a 10 μl PCR reaction with 1μl template DNA. The PCR thermocycling protocol included the following steps: 10 min at 95°C followed by 29 cycles of 30 s at 94°C, 30 s at 56/57/58/59°C (annealing temperature differed among multiplex sets, see [11]), 1 min at 72°C, with a final elongation step of 45 min at 72°C. An ABI PRISM 3730 sequencer was used to analyse samples and the GeneMapper v4.1 (Applied Biosystems) software program was used for subsequent genotyping. For assessment of genotyping reliability, 10% of the samples were chosen randomly and analysed a second time.

### Genetic summary statistics

We used Micro-Checker 2.2.3 [56] to detect the potential presence of null alleles and scoring errors due to large dropout alleles or stutter. Tests for linkage disequilibrium and deviations from Hardy-Weinberg expectations were carried out both at a genetic cluster and geographic region level in Genepop 4.7.0 [57] with the following parameter settings: dememorization = 10,000, batches = 5,000, and iterations per batch = 10,000. We calculated the inbreeding coefficient (F_IS_) as well as significance levels with FSTAT 2.9.3.2 [36–38]. Finally, GenAlEx 6.51b2 [47, 58] was used to estimate genetic summary statistics (i.e., observed and expected heterozygosity,) for all groups analysed.

### Population genetic structure

We applied a Bayesian clustering algorithm using the software package STRUCTURE 2.3.4 [43, 59] to assess overall and sex-specific genetic clustering. The entire dataset of 369 individuals was initially used, without any pre-defined populations, to obtain the most reliable assignment scores possible. These assignment scores were subsequently utilized for sorting individuals into genetic clusters and admixed groups after removal of 0-year olds to limit the analysis of sex-biased dispersal to adult specimens in subsequent analyses. An assignment score of q < 0.7 was used to identify genotypes with admixed ancestry. Using the correlated allele frequency model and assuming admixture, forty independent runs of K = 1–10 were carried out with a burn-in of 100,000 and 1,000,000 MCMC repetitions. The runs were conducted on the CIPRES Portal v3.3 at the San Diego Supercomputer Center (https://www.phylo.org/; [60]) that permits parallelised computation with the R package PARALLELSTRUCTURE [61] to reduce computation time. We processed STRUCTURE results with CLUMPAK [62]. Following [63], the modal value of the ad hoc quantity ΔK was used as the criterion to infer the most likely number of genetic clusters. For preparation of ΔK graphics, only K = 1–5 were used to save space and to make them comparable to previous analyses [11]. However, full analytical details and additional ΔK and STRUCTURE bar plots based on K = 1–10 can be found in S2 and S3 in S1 Material.

A second set of analyses served as an additional test to determine the most likely number of K and was based on pre-defined sub-populations along the distribution continuum and along the recolonization axis from east to west. We did this by pooling the individuals according to their sampling location into three regions; western, central, and eastern; each containing 1/3 of the samples (Fig 1), which matched the cluster-based analysis closely and therefore, allowed for comparison of statistical results retrieved from the two different groupings. Since we had pre-defined populations in this case, we ran these analyses twice, once without and once using the LOCPRIOR function; the latter incorporates spatial information as a prior in the model. With the LOCPRIOR, the clustering algorithm assumes that a probability of an individual to be assigned to a cluster varies according to its sampling location, decreasing the potential of finding false population structuring [64]. We sorted all individuals in all STRUCTURE bar plots from west to east based on georeferences (i.e., latitude/longitude) to make any geographical pattern easier to identify.

Finally, because unequal sample sizes occurred in the geographical STRUCTURE analyses, we accounted for that by determining the most likely number of K with STRUCTURESELECTOR [65].

### Spatial autocorrelation and Principal Coordinate Analysis (PCoA)

We performed spatial autocorrelation analyses using GenAlEx 6.51b2 with 9,999 permutations [47, 58] to compare the pairwise relationship between genetic and spatial distance between males and females. We used 15 km distance class for both sexes, because tests of other distances classes (i.e., 5, 10, and 20 km; results not shown) gave nearly identical results for males and because 15 km was already determined to be appropriate for females in [11]. Because pooling data from genetically differentiated clusters may potentially bias the overall spatial autocorrelation coefficient, *r*, we ran the analysis on the regional level (all genotypes within each sex) using the multiple genetic cluster approach [11], treating the detected genetic clusters and admixed individuals as groups. Additionally, we also ran the analysis separately for male and female genotypes both within clusters assigned by STRUCTURE and by dividing the data set into three geographical regions to conclude whether the possible bias is spatially variable. For the interpretation of the results, we considered both overall significance (following recommendations by [47, 58], we used an ɑ = 0.01) and whether the 95% confidence intervals (CI) were above an r = 0 to avoid false positive results.

In addition, we performed a Principal Coordinate Analysis (PCoA) for all samples, divided by sex, with GenAlEx 6.51b2 [47, 58]. Standardized covariances of genetic distances were used in this analysis.

### Analyses of molecular variance

We performed an AMOVA (Analyses of Molecular Variance, [42]) to investigate differences between sexes in levels of genetic variation partitioning among and within both STRUCTURE clusters and the three geographical groups as described above to test whether the exclusion of admixed individuals and first-generation migrants or spatial overlap of clusters affected our results.

### Assignment indices and genetic diversity estimators

We assessed differences between sexes in five genetic diversity estimators (i.e., Relatedness (r), genetic differentiation (F_ST_), inbreeding coefficient (F_IS_), mean (mAIc) and variance (vAIc) of the corrected assignment index AI (AIc) with the software package FSTAT 2.9.3.2 [35–38] and tested for significance using a permutation test with 5,000 iterations. In addition, we also estimated AIc and mAIc based on the methodology by [37] and extended by [66]. Significance of mAIc was determined with a nonparametric Mann Whitney U-test (MW-U) in GenAlEx 6.51b2 [47, 58]. All tests were run twice; first on the genetic clusters as retrieved by the STRUCTURE analysis and second, on individuals pooled according to the predefined geographical regions.

## Results

### Genotyping and genetic variation

Genetic variation was found to be relatively high for the population (n = 282) with average observed and expected heterozygosity of 0.651 (standard error: 0.03) and 0.662 (standard error: 0.03) Accordingly, only one locus (i.e., Fca-077) showed a highly significant F_IS_ estimate (S1 Table in S1 Material) and no large differences between observed and expected heterozygosity either at the locus level or at the genetic cluster and geographical group level was observed (S1 and S2 Tables in S1 Material). There was no indication of scoring errors due to stutter or large allele dropout. Only one locus showed significant evidence for null alleles (Fca-077 in the eastern geographical group EAST) and therefore, overall evidence for null alleles was considered low. All loci were polymorphic and in Hardy-Weinberg equilibrium after Bonferroni correction, except Fca-077 in (eastern) geographical region EAST and genetic clusters 1 and 2. Based on geographical regions, no loci pairs were in linkage disequilibrium after Bonferroni correction for multiple testing (90/630 pairwise comparisons were significant at P = 0.05 level). At the genetic cluster level, 84/630 tests were significant at the 0.05 level, of which three remained significant after Bonferroni correction, but none of those were observed for the same STR combinations across all three clusters (i.e., Fca90—Fca723 and Lc106—Fca126 in cluster 2 and Fca90—Fca723 in the admixed group). Since both tests did not show a consistent pattern of any loci pairs being in linkage disequilibrium, all loci were retained for further analysis.

### Population genetic structure

**Fig 2:**
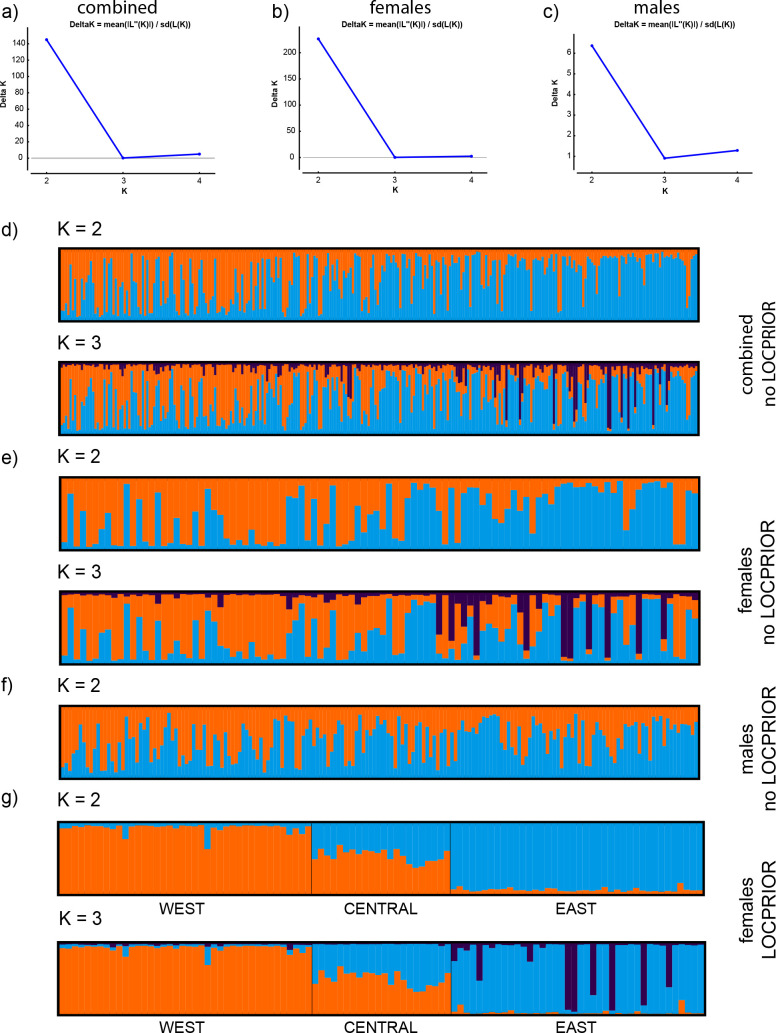
Spatial genetic structure identified by Bayesian cluster assignment analysis for Eurasian lynx in southern Finland. A-C show the DeltaK plots for (A) the combined data set, (B) females, and (C) males, respectively. (D-G) CLUMPAK-averaged Bayesian clustering (STRUCTURE) plots showing posterior probabilities of lynx individual genotypes (as bars) assigned to each genetic cluster based on STR data for K = 2–3. Individuals are sorted by geography from west to east in STRUCTURE bar plots. (D) sexes combined (N = 282), 2E) females (N = 102), and 2F) males (N = 180). In addition, (G) shows the bar plot for females across three geographical regions as retrieved from a STRUCTURE run with the LOCPRIOR option.

For all three data sets (i.e., sexes combined, females and males), two distinct genetic clusters were identified by the Evanno method (ΔK = 2) when running the analysis without predefined populations (Fig 2A–2C; S2A–S2C Table in S1 Material; [43, 64]).

For females, the clusters displayed a gradual shift from west to east in relative distribution across the study area (Figs 1, 2E and 2G). In contrast, males showed complete spatial admixture (Fig 2F). For the combined data set, including both sexes, this pattern was present, but weaker (Fig 2D).

Although a model of three genetic clusters received low statistical support, the STRUCTURE bar plots indicated the existence of a third genetic cluster in females, as indicated by individuals with high assignment scores to a third (purple) cluster, located in the eastern part of the study area (Fig 2E and 2G; S3K–S3L Fig in S1 Material). The STRUCTURE analyses based on geographical regions either using the LOCPRIOR function or not was consistent with this interpretation when accounting for unequal sample sizes, supporting differential genetic structuring between sexes (Fig 2G; S3 Material in S1 Material).

### Spatial autocorrelation and Principal Coordinate Analysis (PCoA)

**Fig 3:**
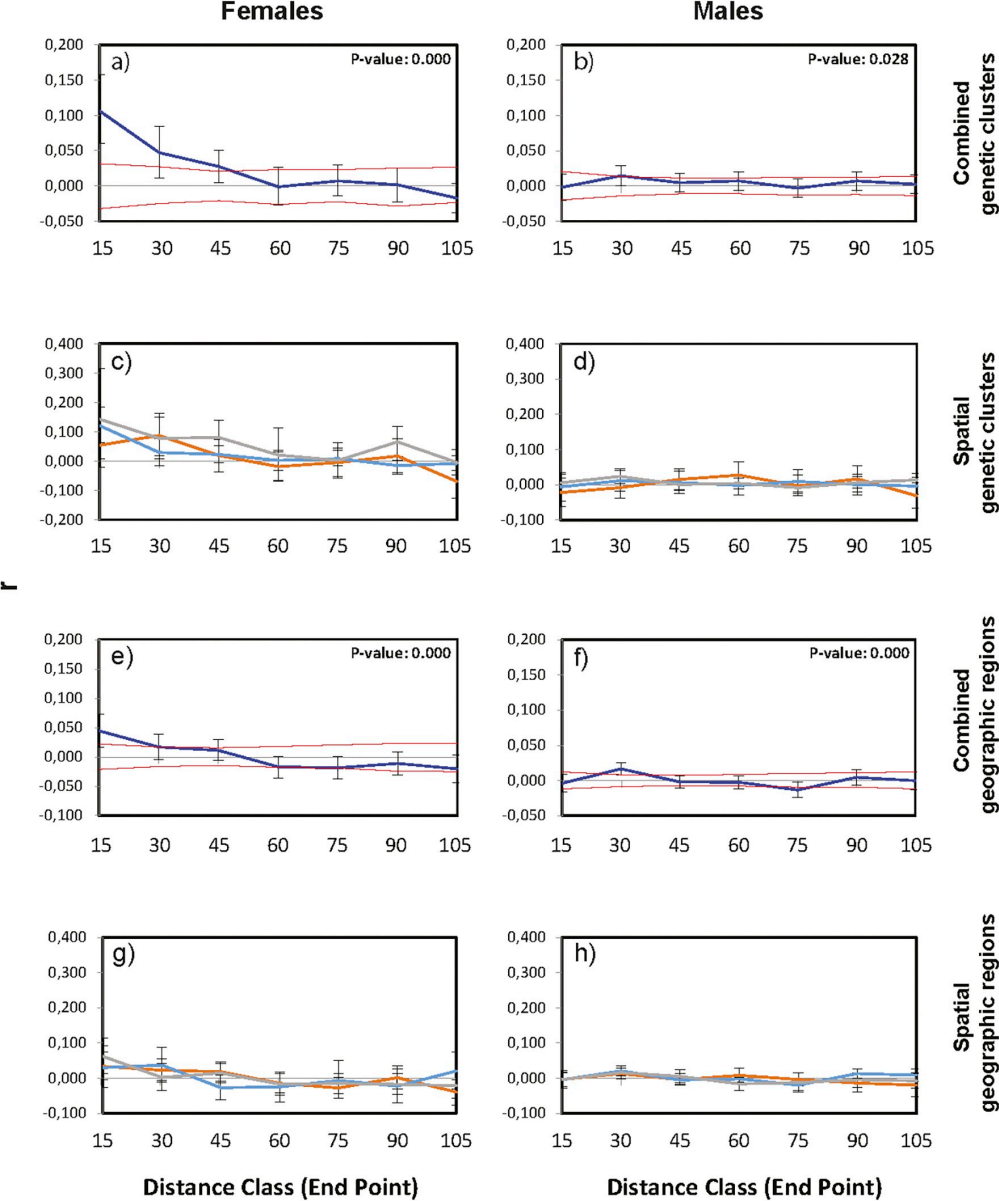
Spatial autocorrelation within geographic distance classes for genetic clusters and geographical groups. (A) Combined spatial structure analysis for females in both genetic clusters and the admixed individuals. (B) Combined spatial structure analysis for males in both genetic clusters and the admixed individuals. (C) Spatial autocorrelation for females in both genetic clusters and admixed individuals. (D) Spatial autocorrelation for females in both genetic clusters and admixed individuals. Genetic cluster 1 = orange line, genetic cluster 2 = blue line, admixed individuals = grey line. F = females, M = males. 3E) Combined spatial structure analysis for females based on geographical regions. 3F) Combined spatial structure analysis for males based on geographical regions. 3G) Spatial autocorrelation for females in the three geographical regions. 3H) Spatial autocorrelation for males in the three geographical regions: WEST = orange line, CENTRAL = blue line, and EAST = grey line. The 95% confidence interval for the null hypothesis of random distribution is given as a dashed line, the bootstrap errors are displayed as whiskers.

Across genetic clusters, females showed significant decreasing spatial autocorrelation coefficient, r, up to 45 km (r_females_ = 0.106, P value: 0.000; Fig 3A), whereas males did not show decreasing spatial autocorrelation (r_males_ = -0.002; P value: 0.028; Fig 3B). This difference between the sexes was apparent also within genetic clusters (Fig 3C and 3D), although non-significant for females in cluster 1 (r_cluster1_ = 0.055; P value: 0.001, but error bars were regularly below r = 0; r_cluster2_ = 0.121, P value: 0.000; r_admixed_ = 0.142, P value: 0.001; Fig 3C). Males showed no spatial autocorrelation in any of the genetic clusters (r_cluster1_ = -0.022, P value: 0.017, r_cluster2_ = -0.006, P value: 0.363, r_admixed_ = 0.006, P value: 0.008, but error bars were regularly below r = 0; Fig 3D). Across geographic regions, a similar pattern emerged; however, the estimated sex-bias was reduced with more than 50% compared to the cluster-based analysis (r_females_ = 0034, P value = 0.000; r_males_ = -0.004, P value = 0.000, but error bars were regularly below r = 0; Fig 3E and 3F). Similarly, within geographical regions, females displayed consistently lower spatial autocorrelation estimates (r_WEST_ = 0.034, P value = 0.001, but error bars were regularly below r = 0; r_CENTRAL_ = 0.030, P value = 0.011, r_EAST_ = 0.062, P value = 0.006; Fig 3G) than within genetic clusters, while males again showed no spatial autocorrelation at all (r_WEST_ = -0.003, P value = 0.034; r_CENTRAL_ = -0.004, P value = 0.000, but error bars were regularly below r = 0; r_EAST_ = -0.004, P value = 0.019; Fig 3H). Although differences in sample size of the different approaches, may have had an impact on estimates in the spatial autocorrelation analyses, the consistency of the weaker pattern in the geographical region analysis makes a random effect unlikely.

The PCoA revealed no clear difference between the sexes (S4 Fig in S1 Material). However, the genotypes that were assigned to a third genetic cluster in the STRUCTURE analysis (Fig 2E and 2G) were situated at the periphery of the scatter plot, confirming their distinctiveness.

### Analyses of molecular variance

When comparing the molecular variance among both identified genetic clusters and the group of admixed individuals in an AMOVA analysis (Table 2), significant and similar between-cluster differentiation was found both overall and in females and males separately. In contrast, the analysis based on geographical regions gave similar results as the sex-specific STRUCTURE analysis (Table 2) in that lower partitioning of molecular variance across spatial groups was found in males than in females, consistent with male-biased dispersal.

**Table 2:** Analysis of Molecular variance (AMOVA) among genetic clusters and geographical regions for the Eurasian lynx in southern Finland 2003–2017.

	Sex	Between-sex variation (%)	Between-group variation (%)	F_ST_	P
**Genetic clusters 1, 2 and admixed**	F	-	3.0	0.031	**0.000**
	M	-	3.0	0.026	**0.000**
	F+M	0	3.0	0.018	**0.000**
**Genetic clusters 1 and 2**	F	-	5.0	0.050	**0.000**
	M	-	6.0	0.056	**0.000**
	F+M	0	5.0	0.027	**0.000**
**Geographical regions WEST, CENTRAL, EAST**	F	-	2.0	0.022	**0.000**
	M	-	0	0.003	**0.018**
	F+M	0	1.0	0.010	**0.000**

STRUCTURE genetic clusters (CL1 = 75 individuals and CL2 = 102 individuals), and admixed (105 individuals). Likewise, an AMOVA was conducted for the three geographical regions (WEST, CENTRAL, and EAST) with 94 individuals in each of the groups. To test for sex-specific patterns, the analysis was performed also for both sexes (F = females, M = males) separately. Significant results in bold.

### Assignment indices

**Fig 4:**
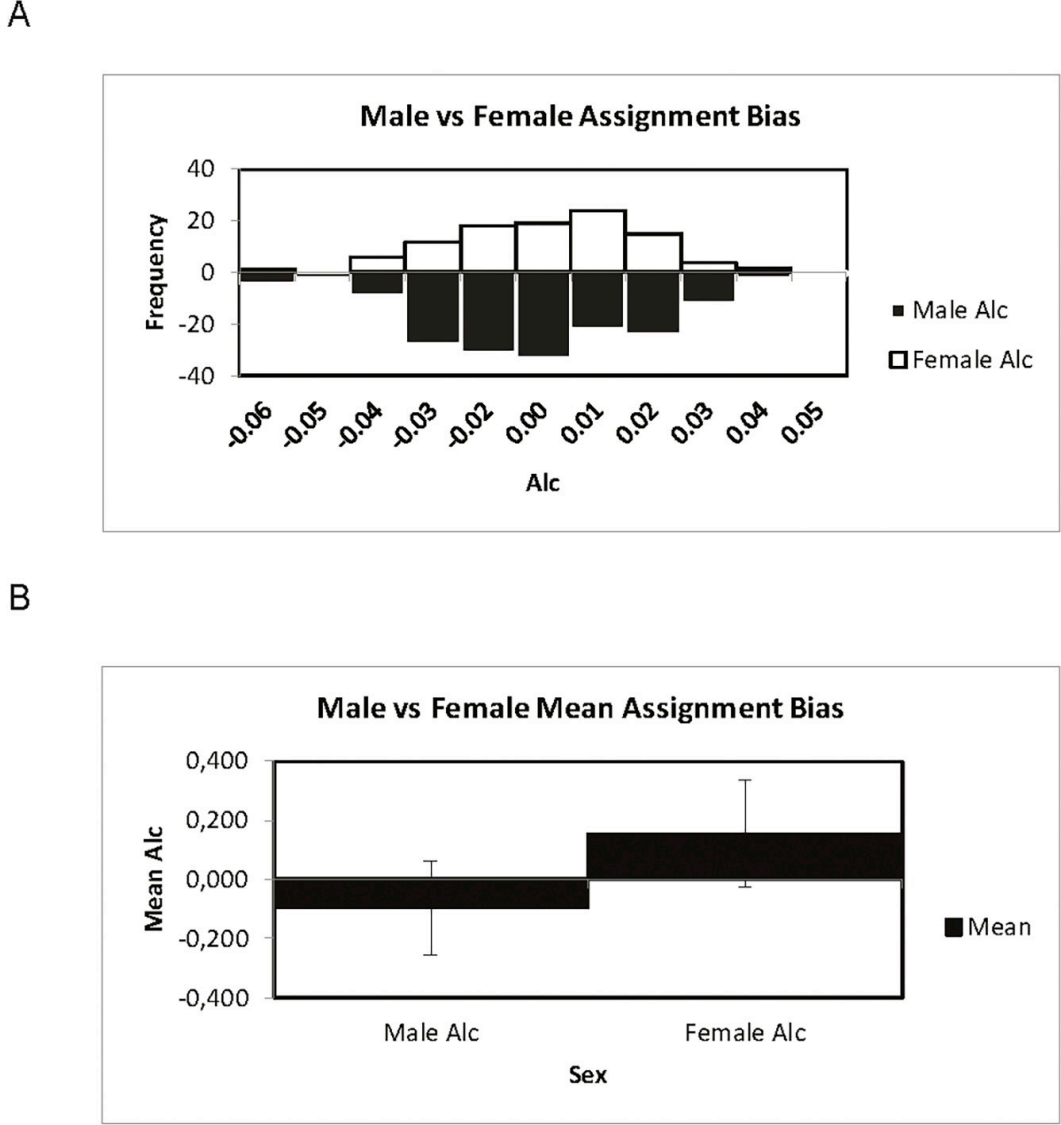
Frequency distribution and mean of corrected genetic assignment indices (AIc) for lynx males and females. (A) Frequency distribution of AIc (corrected Assignment Index) values in the entire data set. Data for males shown in black bars and data for females shown in white bars. (B) Mean AIc values in males and females.

The results from FSTAT analyses showed significantly lower mAIc (mean Assignment Indices corrected) values with higher variance (i.e., vAIc, Table 3) in males than females. However, the difference between sexes was larger in genetic cluster-based analysis than in geographical region-based analyses (Table 3). Consistent with this, taking out female individuals that grouped in a third separate cluster only found in the eastern part of the study area in the STRUCTURE analysis (Fig 2E) also made the sex-bias clearer (results not shown). Similarly, GENALEX analyses showed a higher frequency of negative corrected assignment indices (AIc-values; Fig 4A) and lower mean AIc-values (Fig 4B) in males than females although the latter difference was not statistically significant unless removing the females that grouped in a third separate cluster (Fig 2E). Limiting the analysis to the eastern cluster 2, the strength of sex-bias increased when removing cluster 3 females although results were non-significant (results not shown).

**Table 3:** Genetic FSTAT statistic among genetic clusters and geographical regions for the Eurasian lynx in southern Finland 2003–2017.

Entire range: Genetic clusters 1, 2 and admixed	mAIc	vAIc	F_ST_	F_IS_	REL	H_0_	H_S_
**Females**	0.948	15.009	0.031	-0.003	0.061	0.646	0.644
**Males**	-0.537	46.144	0.025	0.014	0.048	0.655	0.664
**P value**	0.017	0.001	0.575	0.368	0.546	0.516	**0.012**
**Geographical regions WEST, CENTRAL, and EAST**							
**Females**	0.805	12.244	0.022	0.003	0.042	0.646	0.648
**Males**	-0.456	22.265	0.003	0.017	0.006	0.654	0.665
**P value**	0.005	0.001	0.001	0.275	0.001	0.246	**0.010**

Values for females (N = 102) / males (N = 180) are given followed by the P value. Abbreviations: mAIc = mean Assignment Indices corrected, vAIc = variance Assignment Indices corrected, F_ST_ = genetic differentiation, F_IS_ = inbreeding coefficient, REL = relatedness, H_O_ = observed heterozygosity, H_S_ = within-site gene diversity.

### FSTAT genetic diversity estimators and genetic relatedness

All FSTAT estimators showed outcomes expected for the philopatric and dispersing sex, respectively, albeit only three of the seven estimators were statistically significant at the cluster-based level (i.e., mAIc, vAIc, and H_S_) and two additional estimators at the geographical region level (i.e., F_ST_ and REL, Table 3).

**Fig 5:**
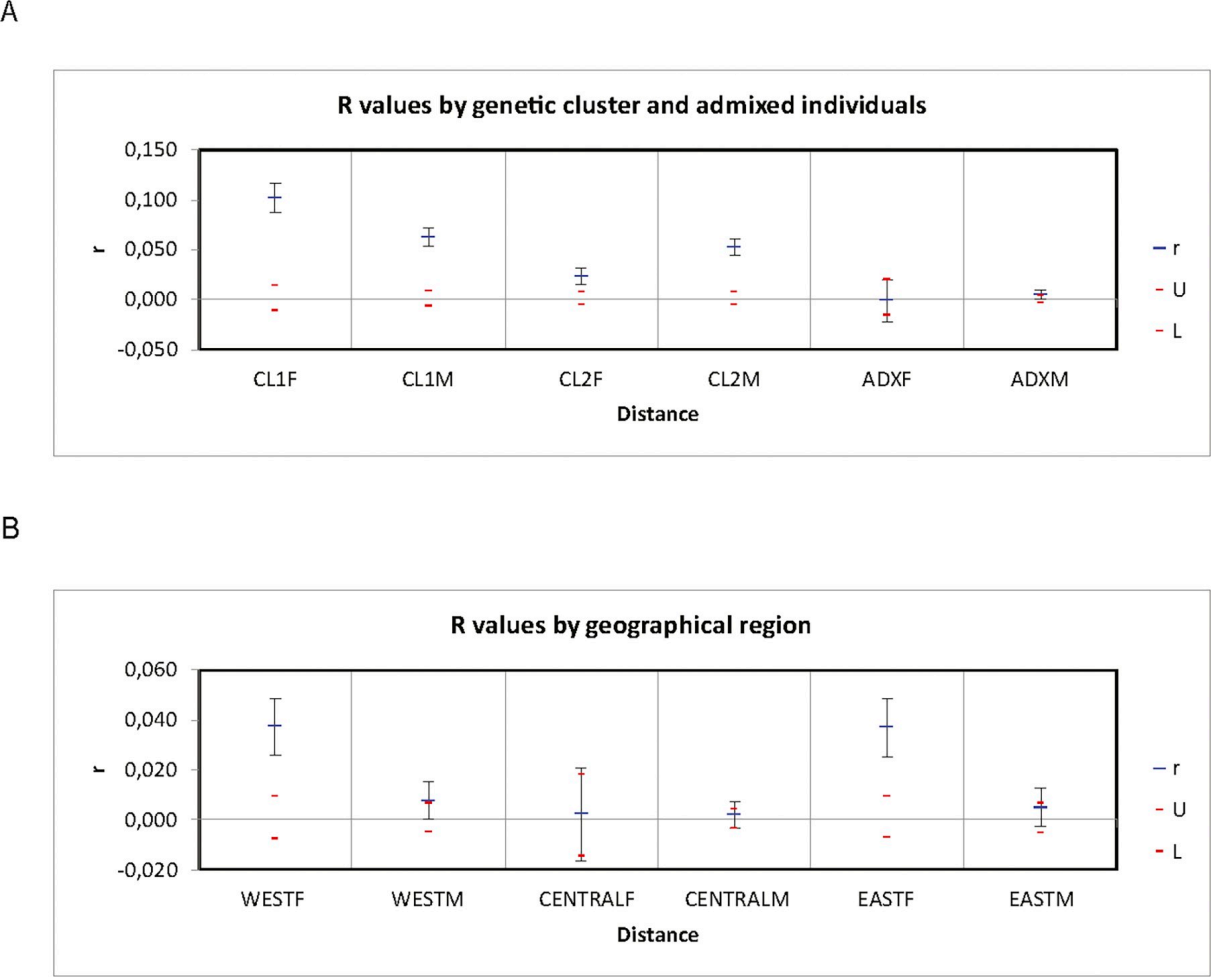
Comparison of lynx relatedness estimates among genetic clusters (A) and geographical regions (B). Abbreviations: CL1F = cluster 1 females, CL2F = cluster 2 females, ADXF = admixed females, CL1M = cluster 1 males, CL2 = cluster 2 males, ADXM = admixed males. WEST = western geographical region, CENTRAL = geographical region situated in the centre of the distribution range, EAST = eastern geographical region. The upper (U) and lower (L) boundaries for the 95% confidence interval for the null hypothesis of random distribution is given as red lines, the bootstrap errors are displayed as whiskers.

Thus, the majority of FSTAT estimators suggested male-biased dispersal, particularly the analysis based on geographical regions. Group-based estimates of relatedness were on average higher when based on genetic clusters than on geographic regions. While the genetic clusters showed no consistent difference in relatedness between sexes, two out of three geographical regions showed significantly higher relatedness among females than males (Fig 5B). The central region (CENTRAL) and the admixed group showed the lowest average relatedness estimates and no difference in relatedness between sexes.

## Discussion

This study’s genetic data supported a pattern of male-biased dispersal and female philopatry in continuously distributed Eurasian lynx in Southern Finland. However, the strength and significance of this pattern was spatially variable and highly dependent on the statistical method used, chosen population groupings (i.e., genetic cluster or geography), and analysis scale (i.e., population or individual-based).

The observed spatial gradient in sex-biased dispersal indicated regional differences in the spatial organization of female relatives, since no similar pattern was observed for males. Males showed spatial admixture between genetic clusters, while females were spatially structured. Spatial autocorrelation coefficient, r, and relatedness (REL) were also significantly stronger in females than males. However, we found weaker sex-specific patterns when the data were separated in three geographical regions than when divided into the two genetic clusters based on the spatial autocorrelation coefficient, r, and relatedness (REL) obtained in the FSTAT analysis, although the latter was significant based on the group-based analysis in terms of the difference between sexes. Assignment indices were also consistently larger when the analysis was based on genetic clusters than on geographic regions. In contrast, molecular variance showed higher structuring for females for the three geographical regions than for the two genetic clusters. FSTAT estimators supported male-biased dispersal, particularly the analysis based on geographical regions. Thus, the ability to detect sex-biased dispersal was dependent on the analytical methods applied, on whether underlying genetic structuring was considered or not, and the choice of groupings.

### Methodological considerations

Both individual-based methods (i.e., spatial autocorrelation and Bayesian clustering analysis) clearly indicated male dispersal and female philopatry. Although seldom applied to test for sex-biased dispersal, Bayesian individual assignment methods like STRUCTURE may thus reveal sex-specific structure and help define genetic clusters to be used in population-based methods (see also [44, 66]). Similarly, the power of spatial autocorrelation analysis peaks at the scale where clustering of related individuals is the highest. Hence, conducting the spatial autocorrelation analysis based on genetic clusters may have contributed to the detection of sex-biased dispersal. In situations like this, a correction for spatial genetic structure may be advisable to detect subtle sex-biased dispersal patterns for large carnivores and other species that have large home ranges, continuous populations, and for species, in which both sexes disperse to different degrees, as demonstrated here. Our results suggest that the combination of the two individual-based genetic approaches may provide complementary information. Non-spatial methods, like PCoA, did not reveal clear patterns between the sexes, indicating that methods taking into account geographic information are more suitable at small geographic scales.

In addition, we used three different population-based statistical methods. While the AMOVA based on geographical regions indicated higher structuring and philopatry in females than in males, the cluster-based analysis showed similar estimates for both sexes. This suggests that the latter approach may underestimate the sex-bias if the genetic clusters show spatial overlap, since genetic variation between sexes may be geographically distributed more evenly than among genetic clusters. Excluding admixed individuals and first-generation migrants (which are presumably more common in the dispersing sex) in the genetic cluster analysis may also lead to a downwards bias in the molecular variance difference between the sexes. Therefore, AMOVA is probably most effective if applied directly on geographical sampling locations in continuous populations. For the assignment indices, we found that even subtle sex-specific substructure can reduce the power of the analysis for sex-biased dispersal testing. Female individuals from a different genetic cluster located only in part of the study area, lowered the positive assignment index, both overall and particularly in cluster 2/eastern geographical region as those females were identified as immigrants. By excluding those individuals, the dispersal asymmetry increased, which also increased the power of the tests [36]. This bias seems to particularly affect the assignment indices while, for example, relatedness estimates per group were only slightly changed (results not shown) when taking cluster 3 females out of the analyses, indicating that summary statistics may be less prone to the presence of immigrants than assignment indices.

Some tests of sex-biased dispersal, such as F_IS_ and observed heterozygosity, appeared less powerful in our study independent of the groupings assessed. This result is consistent with tests by [36], who further showed that F_ST_ and relatedness perform particularly well when the proportion of dispersers is high in the sample, which is facilitated by using a geographical region approach rather than a genetic cluster approach. Estimators like mAIc, vAIc, and H_S_ appear less sensitive to this type of bias and may more readily be applied to genetic clusters identified by Bayesian individual assignment tests. On the contrary, our results suggest that population-level metrices, like F_ST_, heterozygosity, and relatedness may be less powerful in situations where population genetic differentiation is generally very low and proportion of dispersers high, leading to weakly differentiated female and male genotypes among populations. This may be better captured in the geographical region approach in our study, by likely including a higher proportion of dispersers. Estimators like mAIc, vAIc, and H_S_ appear less sensitive to this type of bias and may more readily be applied to genetic clusters identified by Bayesian individual assignment tests.

Finally, to test whether the higher proportion of males in the dataset could have an impact on the results, we subsampled the males and ran two additional analyses with GENALEX with two different male subsets, which resulted in higher female means as expected (results not shown). Since the datasets were initially sorted based on geographical coordinates, the subsampled datasets were generated by deleting every second individual. This ensured that the reduced dataset still had a relatively even geographical distribution. Therefore, we consider a potential sampling effect caused by the higher number of males as negligible.

### Biological considerations

While earlier telemetry studies of Eurasian lynx reported conflicting evidence of sex-biased dispersal, we consistently found male-biased dispersal, which is typical for mammals [6–8, 67]. The lynx population in southern Finland has increased from 1,100 to 2,700 adult individuals during years 2007 to 2015 [68] and represents a large and continuous population with gene flow from the Russian population [31, 48]. Our study area was characterized by different land uses and levels of urbanization but lacks dispersal barriers for lynx [25]. This finding is also supported by good population health [69], and active gene transfer [31]. Hence, anthropogenic and environmental changes that may trigger changes in sex-biased dispersal in this large Eurasian lynx population may be low compared to other studies.

Lack of dispersal barriers may result in long-distance dispersal of both sexes [70]. On the other hand, short-distance dispersers may be over-represented in the data if the study area does not reach beyond the species’ dispersal capabilities [40, 71]. Our study area that represents only a part of Finland, covers 300 km in west-east and over 100 km in north-south-direction, which is well within the dispersal distances for both sexes [25]. Hence, we do not consider dispersal capabilities to be a major driving force in this study.

While short-distance dispersal may be the ultimate cause of inbreeding avoidance and kin competition, colonization of new patches is likely the cause for long-distance dispersal and hence independent of sex [31, 72, 73]. Using spatial autocorrelation, no evidence of scale-dependency of sex-biased dispersal [46] was observed in our study. However, there was evidence of spatial variation: relatedness values (r) were relatively similar between sexes in genetic cluster 1 and geographic region WEST, in the western part of the study area, but relatively different between genetic cluster 2 and geographic region EAST (both in the east). Since males showed no spatial patterns on the scales studied, the difference observed between the sexes seemed to be due to variation in the spatial organization of related females, suggesting an overall stable pattern with potential regional differences influencing female philopatry.

The recolonization of lynx in Finland took place from east to west mainly before our sampling period, and the population was at its highest in 2014 [68]. In the east, the lynx population was established earlier, was stable, and mostly saturated in the beginning of our sampling period (Holmala *unpublished*). In contrast, in the western part of our study area, the process of lynx recolonization was still ongoing in the beginning of the sampling period, and thus probably did not represent a saturated population with a stable social organization until around 2010 (Holmala *unpublished*). These different colonization stages may explain the observed spatial variation in sex-specific autocorrelation, which was mainly due to spatial variation in the organization of the females caused by differences in population density and resource availability [74, 75]. Eurasian lynx shows large variation in dispersal distances, with both short and long-distance dispersal for both sexes [21–23, 25, 76]. In a dispersal model, competition for local mates strongly pressures males to disperse in polygynous species, whereas females only disperse if local resources, such as food and breeding sites are limited [77]. Therefore, females greatly benefit from local knowledge in the natal area, and are discouraged from dispersing [8, 75]. We found female dispersal to be slightly higher in the western than eastern part of our study area, supporting an idea of different phases of lynx population development after early recolonization (Holmala *unpublished*) and the potential founder effects therein. Relatively high, slightly male-biased hunting pressure on lynx in Finland constantly provides vacant territories weakening the competition between males, which could lead to less pressure to disperse [72]. We found no such spatial variation in male dispersal at the scales studied here. For females, hunting creates vacant territories for related females to occupy without the need to disperse further. Therefore, future genetic studies should sample also those females that are not hunted to test if the observed pattern of philopatry may possibly be even stronger.

Ongoing anthropogenic pressures can considerably affect future connectivity and viability of the Eurasian lynx population studied, and more broadly recolonizing large carnivore populations that display sex-biased dispersal. Intensifying urbanization, agriculture, and forestry activities are all factors leading to increased habitat patchiness that are more likely to affect the philopatric sex [77]. In addition, habitat features may differ in their importance for genetic connectivity between females and males [78]. Therefore, management strategies need to consider these differences between the sexes and conserve landscape features facilitating dispersal of the philopatric sex.

If hunting more heavily targets the dispersing sex, it may reduce connectivity and increase genetic population structuring and inbreeding [72, 79]. Male Eurasian lynx have a higher chance of being hunted than females, not only because of their trophy value, but also because of their behaviour [80] and because females are partly protected from hunting in Finland. In our study, males showed no spatial variation in dispersal inferred from genetic data, suggesting that this is currently not an important factor in this large and unfragmented population. Philopatry increases female fitness by, for example, easier recruitment into the local population and increased local knowledge [8, 75]. Therefore, hunting that removes related, breeding females from the local population may counteract the major fitness benefits of philopatry. This may, in turn, lead to reduced population growth because individuals are either forced to choose poorly or because of a discrepancy between true habitat quality and the available cues to perceive it [81]. This, in general, may have negative effects for recolonizing large carnivore populations.

## Conclusions

Research on the Eurasian lynx in the past has shown contradictory evidence concerning sex-biased dispersal. Our results point to flexible dispersal behaviour and spatial organization in female Eurasian lynx, which may reflect the influence of factors such as density, adult turnover rate [23, 24, 71] and resource availability. Given the recent re-colonization history, our results are consistent with a less stable situation in the western part of the study area that may have weakened the signal of dispersal bias between males and females by affecting particularly the female population. In addition, a mixture of anthropogenic activities, such as habitat alteration or hunting, may affect the structuring of individuals and possibly weaken the strength of sex-biased dispersal. However, there is currently little evidence that habitat fragmentation in Finland has had a major impact on lynx dispersal.

We systematically applied different genetic analyses and groupings and recorded the power of detection of sex-biased dispersal. Our results indicate that combining a series of analytical approaches and groupings to evaluate potential biases of estimators is recommended. This may be especially important for species with continuous distribution ranges in which populations cannot readily be delineated and where subtle population genetic structure may be present (e.g., cluster 3 individuals in females in this study). Further, careful consideration should be given on how sampling biases may affect the results.

Considering future research objectives, constructing a pedigree may expand our knowledge about inbreeding avoidance and the prerequisites of philopatry, and the extent to which these behavioural traits drive evolutionary dynamics. Also, the effects of hunting on these features, and more profoundly, the survival of matrilineages and genetic variance in the population needs to be investigated when hunting remains the predominant population management tool.

## Supporting information

S1 Material (DOCX)
