# Supplementary material for: Genetic analysis indicates spatial-dependent patterns of sex-biased dispersal in Eurasian lynx in Finland
Source: PLoS One. 2021 Feb 19;16(2):e0246833. doi: 10.1371/journal.pone.0246833 (PMC7894887; doi:10.1371/journal.pone.0246833)
Supplement: S1 Material — (DOCX) [file pone.0246833.s001.docx]

**S1 Material**

**PONE-D-20-20195**

**Genetic analysis indicates spatial-dependent patterns of sex-biased dispersal in Eurasian lynx in Finland**

**Authors**: Annika Herrero, Cornelya F. C. Klütsch, Katja Holmala, Simo N. Maduna, Alexander Kopatz, Hans Geir Eiken, Snorre B. Hagen

**Table S1. Genetic diversity per locus of Eurasian Lynx (*L. lynx*) in southern Finland (N = 282).** Abbreviations: N_G_ = number of genotypes, N_A_ = number of different alleles per locus, H_O_ = observed heterozygosity, H_E_ = expected heterozygosity, F_IS_ = inbreeding coefficient [38], P values = P value for inbreeding coefficient F_IS_. (adjusted P = 0.002 based on 420 randomizations, [57]) using the software FSTAT. Significant results in bold.

| **Locus** | **N_G_** | **N_A_** | **Ho** | **He** | **F_I_** | **P value** |
| --- | --- | --- | --- | --- | --- | --- |
| **Fca90** | 282 | 4 | 0.564 | 0.604 | 0.068 | 0.043 |
| **Fca723** | 282 | 7 | 0.603 | 0.582 | -0.035 | 0.836 |
| **Fca082** | 282 | 5 | 0.706 | 0.732 | 0.038 | 0.162 |
| **Fca149** | 282 | 3 | 0.340 | 0.346 | 0.018 | 0.417 |
| **Fca567** | 282 | 6 | 0.748 | 0.755 | 0.010 | 0.395 |
| **Fca026** | 282 | 7 | 0.681 | 0.716 | 0.050 | 0.069 |
| **Fca031** | 281 | 8 | 0.758 | 0.798 | 0.052 | 0.050 |
| **Fca043** | 282 | 5 | 0.748 | 0.732 | -0.021 | 0.781 |
| **Fca045** | 282 | 4 | 0.316 | 0.316 | 0.003 | 0.502 |
| **F115** | 282 | 12 | 0.773 | 0.826 | 0.066 | 0.012 |
| **Fca008** | 282 | 4 | 0.730 | 0.710 | -0.027 | 0.779 |
| **Lc106** | 282 | 5 | 0.745 | 0.743 | -0.001 | 0.541 |
| **Lc109** | 274 | 8 | 0.799 | 0.795 | -0.004 | 0.607 |
| **Fca126** | 281 | 8 | 0.758 | 0.720 | -0.051 | 0.948 |
| **Fca391** | 282 | 3 | 0.546 | 0.554 | 0.016 | 0.402 |
| **Fca275** | 281 | 8 | 0.804 | 0.786 | -0.021 | 0.781 |
| **Fca293** | 281 | 3 | 0.591 | 0.594 | 0.008 | 0.471 |
| **Fca559** | 279 | 7 | 0.649 | 0.678 | 0.046 | 0.091 |
| **Lc110** | 282 | 5 | 0.582 | 0.594 | 0.023 | 0.331 |
| **Fca123** | 281 | 6 | 0.705 | 0.710 | 0.009 | 0.443 |
| **Fca077** | 282 | 6 | 0.525 | 0.615 | 0.149 | **0.002** |
| **Mean** |  |  | 0.651 | 0.662 | 0.019 |  |
| **SD** |  |  | 0.030 | 0.030 | 0.015 |  |

**S2**. Summary statistics to determine the most likely number of K for STRUCTURE bar plots shown in Figure 2 in the main manuscript. Table 2a) All samples combined; Table 2b) Females; Table 2c) Males.

Table 2a)

| **# K** | **Replications** | **Mean LnP(K)** | **Stdev LnP(K)** | **Ln'(K)** | **\|Ln''(K)\|** | **Delta K** |
| --- | --- | --- | --- | --- | --- | --- |
| **1** | 40 | -20448.87 | 0.19 | NA | NA | NA |
| **2** | 40 | -20147.97 | 1.03 | 300.90 | 170.47 | 165.69 |
| **3** | 40 | -20017.54 | 4.31 | 130.44 | 24.84 | 5.77 |
| **4** | 40 | -19911.94 | 4.48 | 105.60 | 19.51 | 4.35 |
| **5** | 40 | -19825.86 | 39.62 | 86.09 | 7.10 | 0.18 |
| **6** | 40 | -19746.87 | 88.97 | 78.99 | 259.83 | 2.92 |
| **7** | 40 | -19927.71 | 1940.86 | -180.84 | 5862.63 | 3.02 |
| **8** | 40 | -25971.18 | 35170.56 | -6043.48 | 8968.28 | 0.25 |
| **9** | 40 | -40982.94 | 60585.92 | -15011.76 | 20327.55 | 0.34 |
| **10** | 40 | -35667.15 | 55385.68 | 5315.79 | NA | NA |

**Table 2b)**

| **# K** | **Replications** | **Mean LnP(K)** | **Stdev LnP(K)** | **Ln'(K)** | **\|Ln''(K)\|** | **Delta K** |
| --- | --- | --- | --- | --- | --- | --- |
| **1** | **40** | **-5615.48** | **0.32** | **NA** | **NA** | **NA** |
| **2** | **40** | **-5536.18** | **2.11** | **79.31** | **471.38** | **222.99** |
| **3** | **40** | **-5928.25** | **1787.05** | **-392.07** | **207.81** | **0.12** |
| **4** | **40** | **-6112.52** | **2776.80** | **-184.27** | **1288.81** | **0.46** |
| **5** | **40** | **-7585.59** | **5174.60** | **-1473.08** | **531.20** | **0.10** |
| **6** | **40** | **-8527.48** | **8071.17** | **-941.88** | **1309.69** | **0.16** |
| **7** | **40** | **-8159.67** | **8623.54** | **367.81** | **1135.13** | **0.13** |
| **8** | **40** | **-6656.73** | **5778.82** | **1502.94** | **1524.96** | **0.26** |
| **9** | **40** | **-6678.75** | **6848.54** | **-22.02** | **1094.34** | **0.16** |
| **10** | **40** | **-5606.43** | **35.86** | **1072.32** | **NA** | **NA** |

**Table 2c)**

| **# K** | **Reps** | **Mean LnP(K)** | **Stdev LnP(K)** | **Ln'(K)** | **\|Ln''(K)\|** | **Delta K** |
| --- | --- | --- | --- | --- | --- | --- |
| **1** | **40** | **-13252.07** | **0.18** | **NA** | **NA** | **NA** |
| **2** | **40** | **-13128.99** | **1.19** | **123.08** | **107.99** | **90.85** |
| **3** | **40** | **-13113.90** | **3.19** | **15.09** | **94.07** | **29.47** |
| **4** | **40** | **-13192.87** | **8.12** | **-78.98** | **1977.13** | **243.46** |
| **5** | **40** | **-15248.98** | **9155.97** | **-2056.11** | **1890.77** | **0.21** |
| **6** | **40** | **-15414.32** | **8504.17** | **-165.34** | **3125.33** | **0.37** |
| **7** | **40** | **-18704.99** | **17787.29** | **-3290.67** | **5450.80** | **0.31** |
| **8** | **40** | **-16544.85** | **16921.66** | **2160.13** | **12754.58** | **0.75** |
| **9** | **40** | **-27139.30** | **34177.46** | **-10594.45** | **13761.76** | **0.40** |
| **10** | **40** | **-23971.99** | **31572.57** | **3167.31** | **NA** | **NA** |

**S3. Additional STRUCTURE results for geographical regions not shown in the main manuscript:**

In addition to the STRUCTURE runs shown in the main manuscript, we provide here additional DeltaK plots, STRUCTURESELECTOR plots for runs with unequal sample sizes, and bar plots based on K = 1 - 10.

**S3a**. DeltaK plot based on K = 1 – 10 for the entire dataset with both females and males included and split up into equally sized groups (N = 94 for each of the groups) *without* using the LOCPRIOR function, suggesting that K = 2 is the most likely number of populations in the data set.


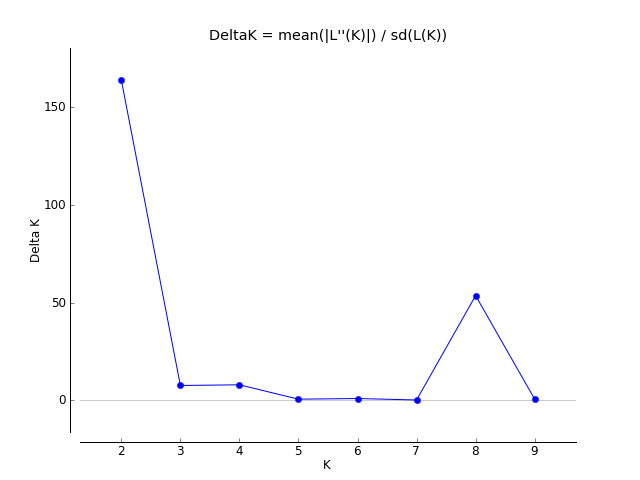


**S3b**. CLUMPAK-averaged Bayesian clustering (STRUCTURE) plots for the entire data set showing posterior probabilities of lynx individual genotypes (as bars) assigned to each genetic cluster based on STR data for K = 2 - 4 for analysis *without* using the LOCPRIOR function. Individuals are sorted by geography from west to east in the STRUCTURE bar plot.


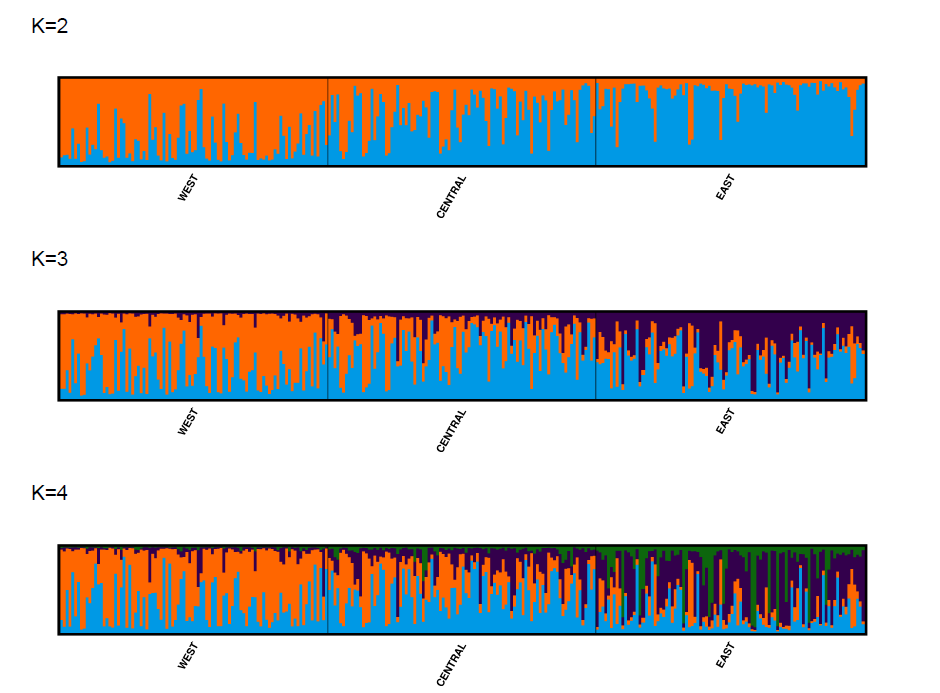


**S3c**. DeltaK plot based on K = 1 - 10for the dataset including females only and split up into three groups with unequal sample sizes (i.e., WEST = 40, CENTRAL = 22, EAST = 40) *without* using the LOCPRIOR function, suggesting that K = 2 is the most likely number of populations in the data set.


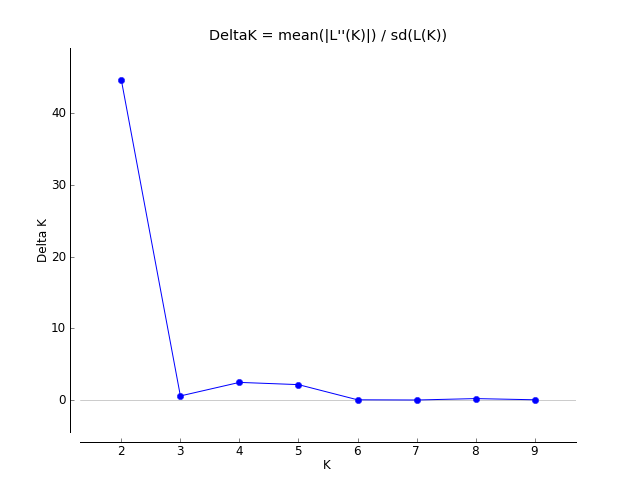


Since the data set for females included groups wof unequal sample sizes, we determined the most likely number of K also with STRUCTURESELECTOR (Li and Liu 2018; Figure S2d). The results indicated in all four estimators that K = 2 is the most likely number of populations.

**S3d**. Estimation of the most likely number of populations for female lynx in southern Finland, conducted with STRUCTURESELECTOR for four recently introduced estimators by Puechmaille (2016): the median of means (MedMeaK), maximum of means (MaxMeaK), median of medians (MedMedK), and maximum of medians (MaxMedK).


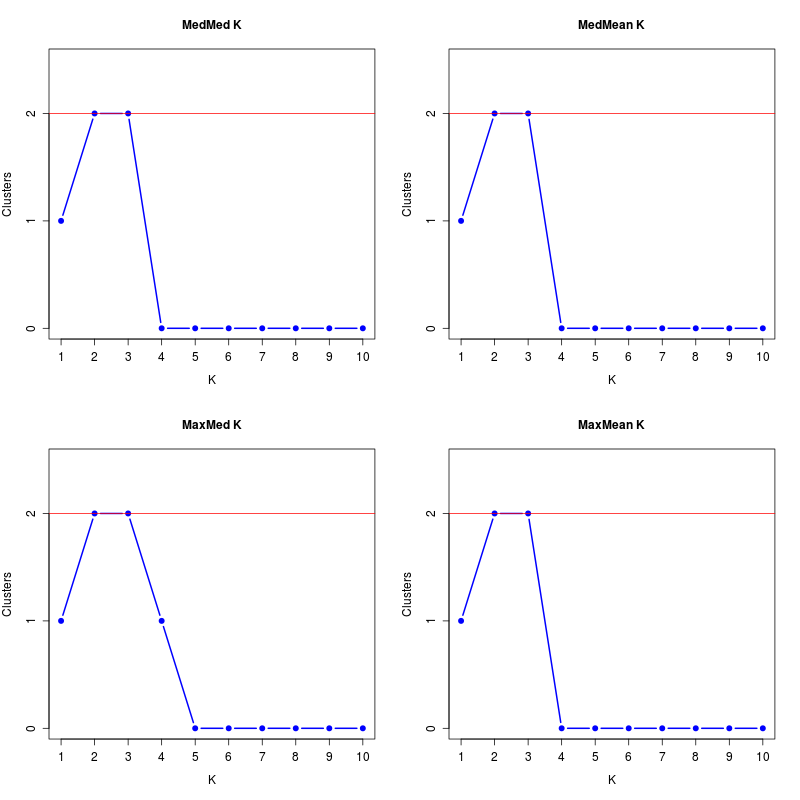


**S3e**. CLUMPAK-averaged Bayesian clustering (STRUCTURE) plots for the data set including females showing posterior probabilities of lynx individual genotypes (as bars) assigned to each genetic cluster based on STR data for K = 2 - 4 for analysis *without* using the LOCPRIOR function. Individuals are sorted by geography from west to east in the STRUCTURE bar plot.


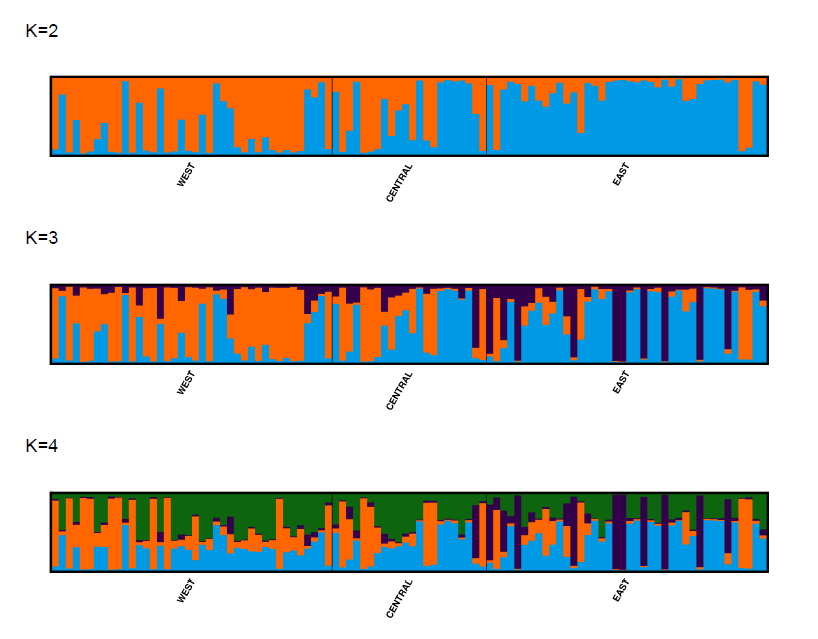


**S3f**. DeltaK plot based on K = 1 - 10for the dataset including males only and split up into three groups with unequal sample sizes (i.e., WEST = 54, CENTRAL = 72, EAST = 54) *without* using the LOCPRIOR function, suggesting that K = 2 is the most likely number of populations in the data set.


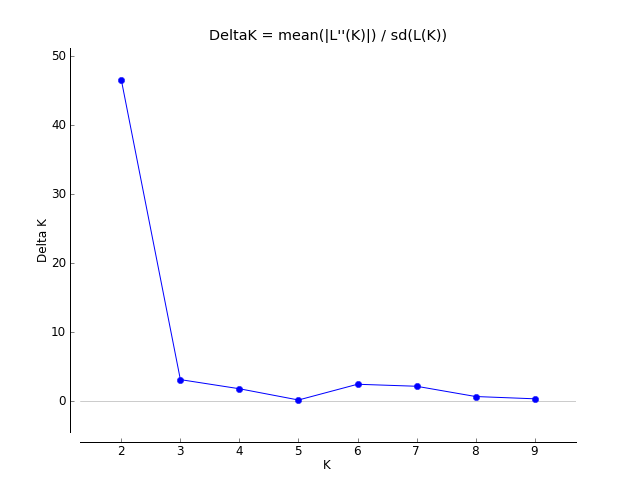


Since the data set for males included groups with unequal sample sizes, we determined the most likely number of K also with STRUCTURESELECTOR (Li and Liu 2018; Figure S2g). The results indicated in all four estimators that K = 2 is the most likely number of populations.

**S3g**. Estimation of the most likely number of populations for male lynx in southern Finland, conducted with STRUCTURESELECTOR for four recently introduced estimators by Puechmaille (2016): the median of means (MedMeaK), maximum of means (MaxMeaK), median of medians (MedMedK), and maximum of medians (MaxMedK).


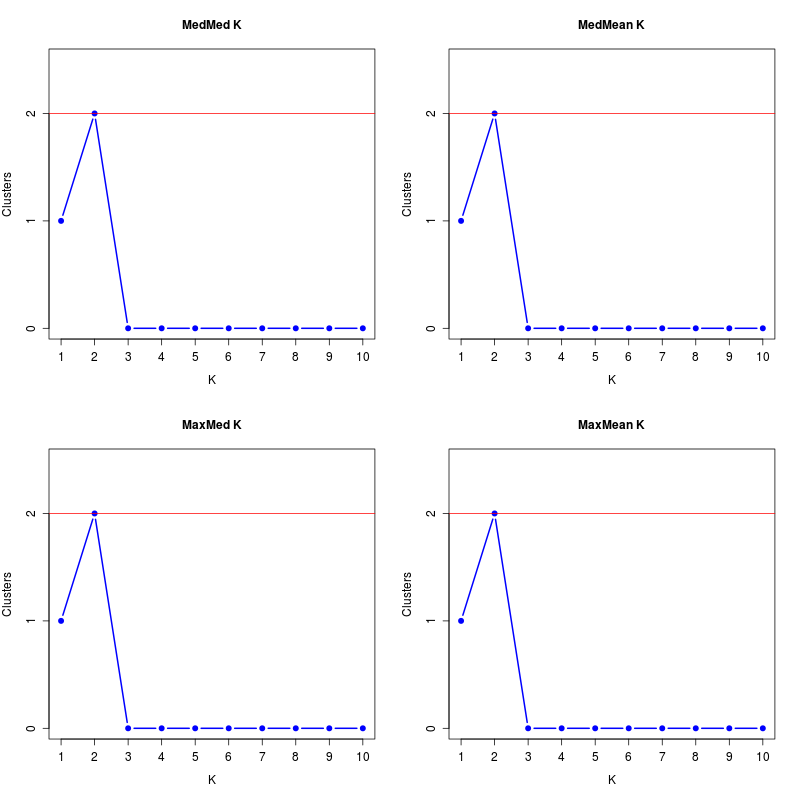


**S3h**. CLUMPAK-averaged Bayesian clustering (STRUCTURE) plots for the data set including males showing posterior probabilities of lynx individual genotypes (as bars) assigned to each genetic cluster based on STR data for K = 2 - 3 for analysis *without* using the LOCPRIOR function. Individuals are sorted by geography from west to east in the STRUCTURE bar plot.


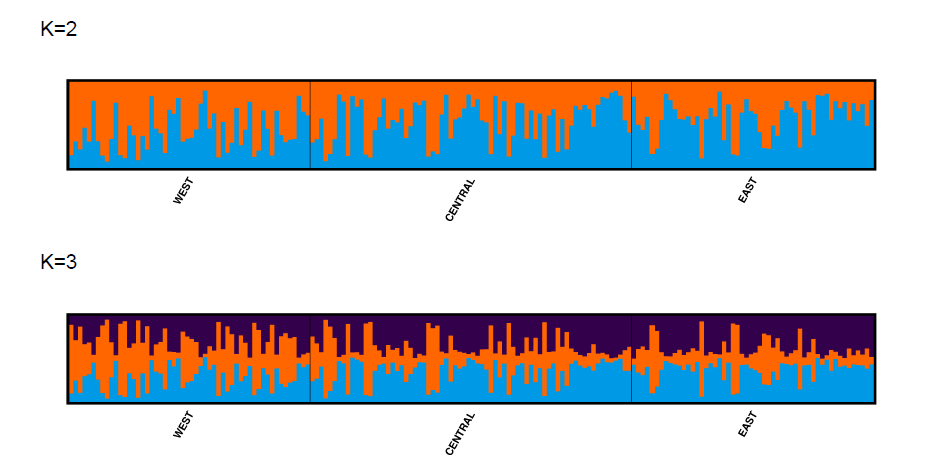


**S3i**. DeltaK plot based on K = 1 - 10for the entire dataset with both females and males included and split up into equally sized groups (N = 94 for each of the groups) using the LOCPRIOR function – DeltaK plot based on ten iterations, suggesting that K = 2 is the most likely number of populations in the data set.


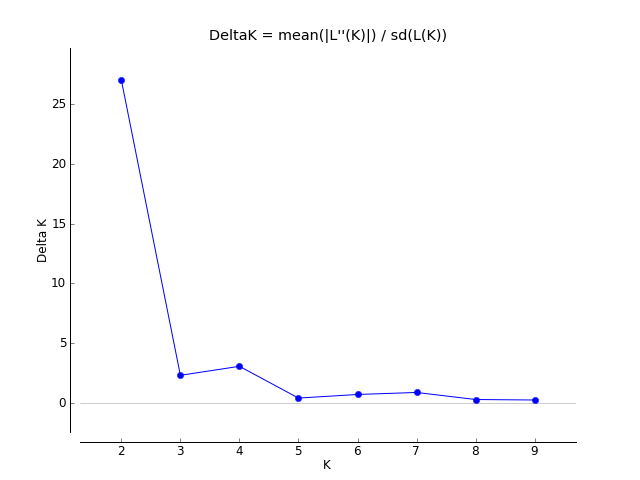


**S3j**. CLUMPAK-averaged Bayesian clustering (STRUCTURE) plots for the entire data set showing posterior probabilities of lynx individual genotypes (as bars) assigned to each genetic cluster based on STR data for K = 2 - 4 for analysis using the LOCPRIOR function. Individuals are sorted by geography from west to east in the STRUCTURE bar plot.


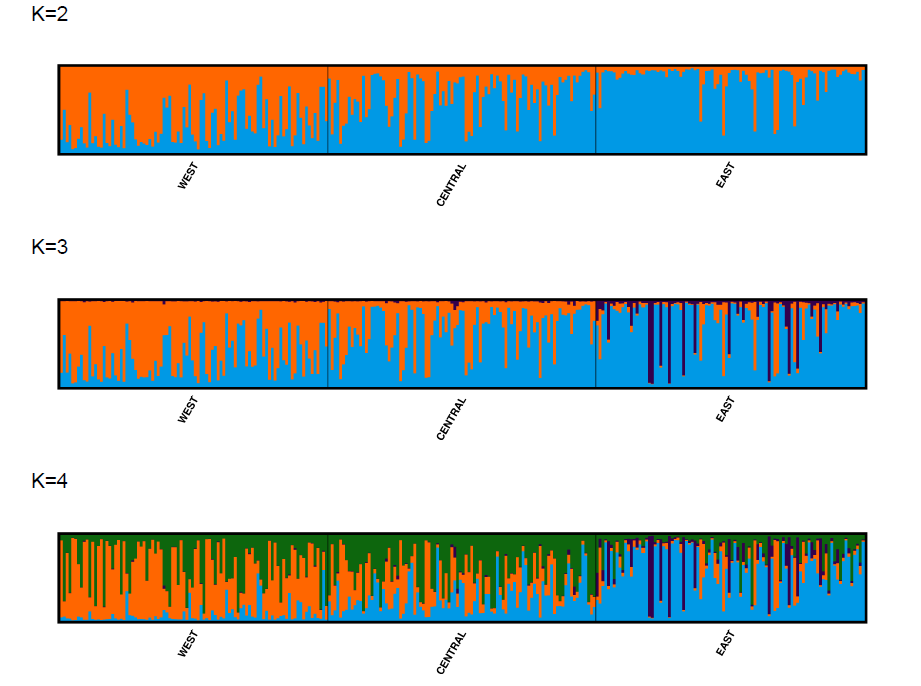


**S3k**. Data set including females only: K = 1 – 10 using the LOCPRIOR function, DeltaK plot, indicating K = 2 -3 to be the most likely number of populations.

*
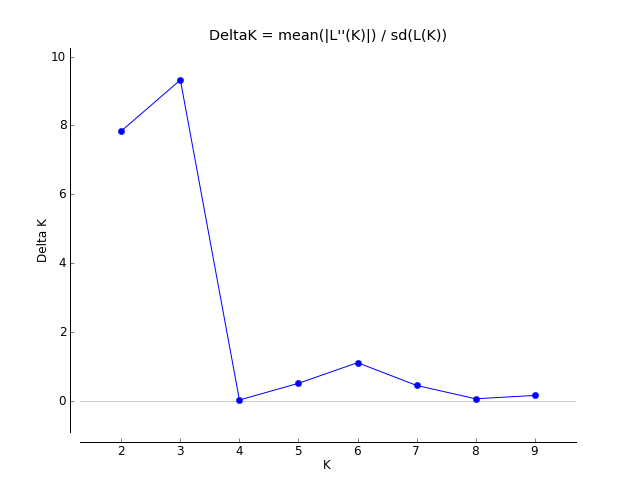
*

In contrast to the entire dataset, that was of equal sample size for all three geographical regions, the female data set consisted of three groups containing different sample sizes (i.e., WEST = 40, CENTRAL = 22, EAST = 40). Since unequal sample sizes can influence the outcomes of Bayesian cluster analyses (Puechmaille 2016), we ran the data set for females also through STRUCTURESELECTOR (Li and Liu 2018; Figure S2l). The results indicated in all four estimators that K = 2 is the most likely number of populations.

**S3l**. Estimation of the most likely number of populations for female lynx in southern Finland, conducted with STRUCTURESELECTOR for four recently introduced estimators by Puechmaille (2016): the median of means (MedMeaK), maximum of means (MaxMeaK), median of medians (MedMedK), and maximum of medians (MaxMedK).


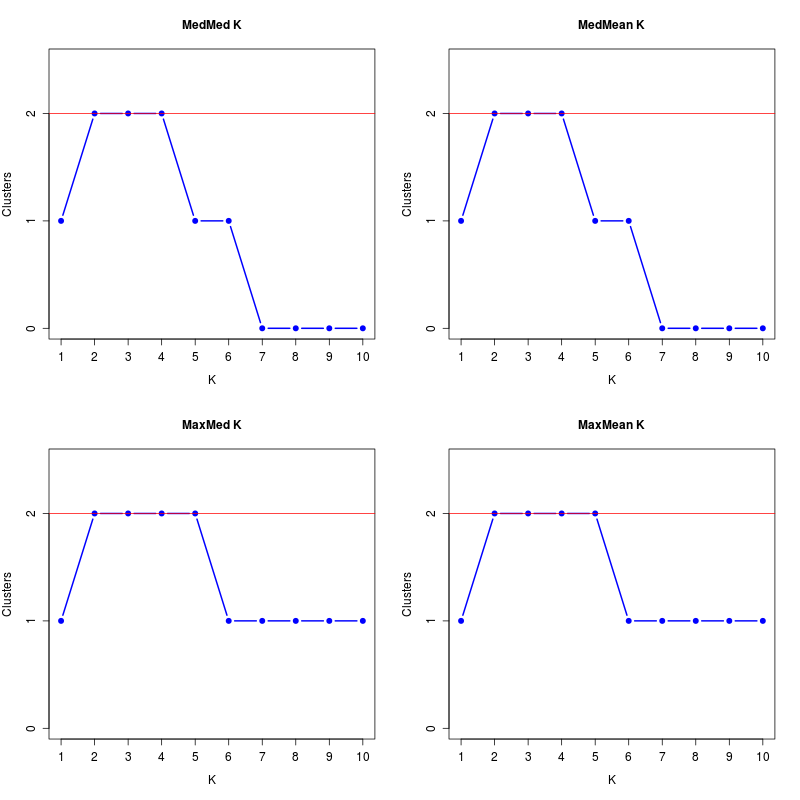


The STRUCTURE bar plot for the dataset for females can be found in the main manuscript (Figure 2j).

**S3m**. Data set including males only: K = 1 – 10 using the LOCPRIOR function, DeltaK plot, indicating K = 2 to be the most likely number of populations.


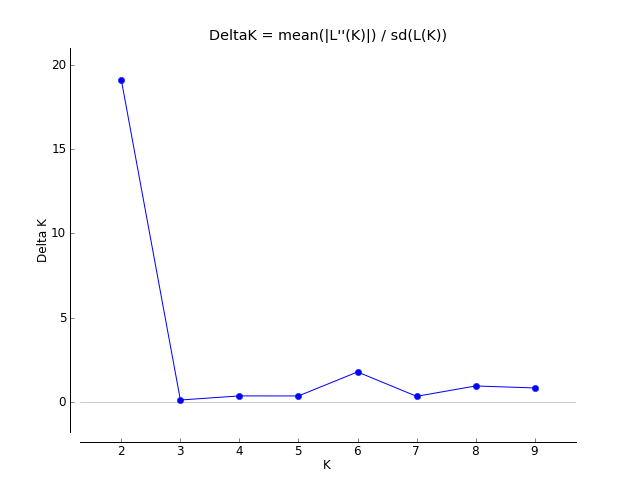


Again, we analyzed the data set for males with STRUCTURESELECTOR (Li and Liu 2018; Figure S2n) due to three groups containing different sample sizes (i.e., WEST = 54, CENTRAL = 72, EAST = 54). The results indicated in all four estimators that K = 2 is the most likely number of populations.

**S3n.** Estimation of the most likely number of populations for male lynx in southern Finland, conducted with STRUCTURESELECTOR for four recently introduced estimators by Puechmaille (2016): the median of means (MedMeaK), maximum of means (MaxMeaK), median of medians (MedMedK), and maximum of medians (MaxMedK).


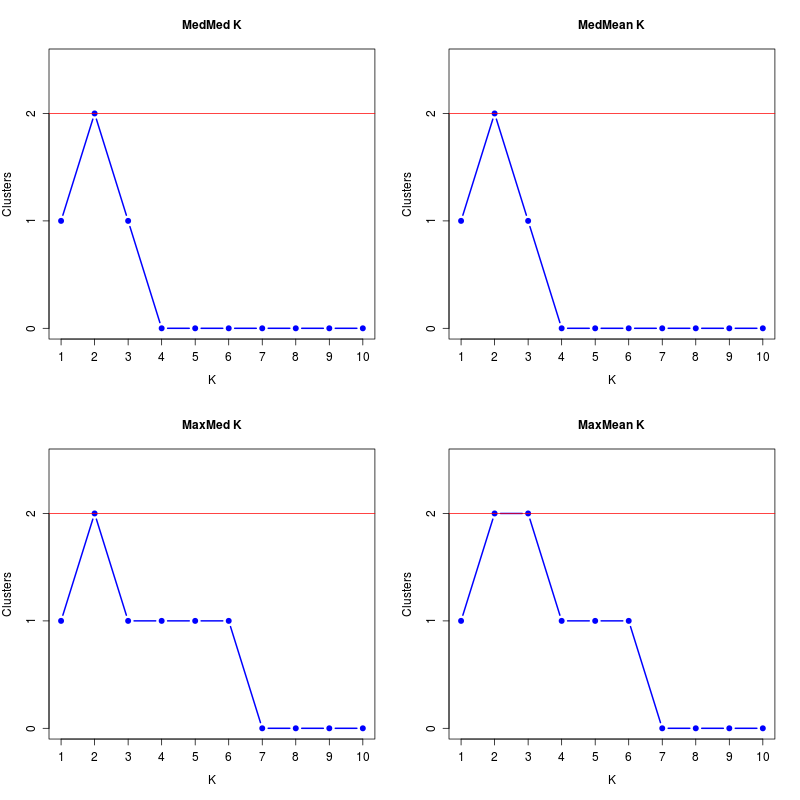


**S3o**. CLUMPAK-averaged Bayesian clustering (STRUCTURE) plots for males showing posterior probabilities of lynx individual genotypes (as bars) assigned to each genetic cluster based on STR data for K = 2 - 3 for analysis using the LOCPRIOR function. Individuals are sorted by geography from west to east in STRUCTURE bar plots.


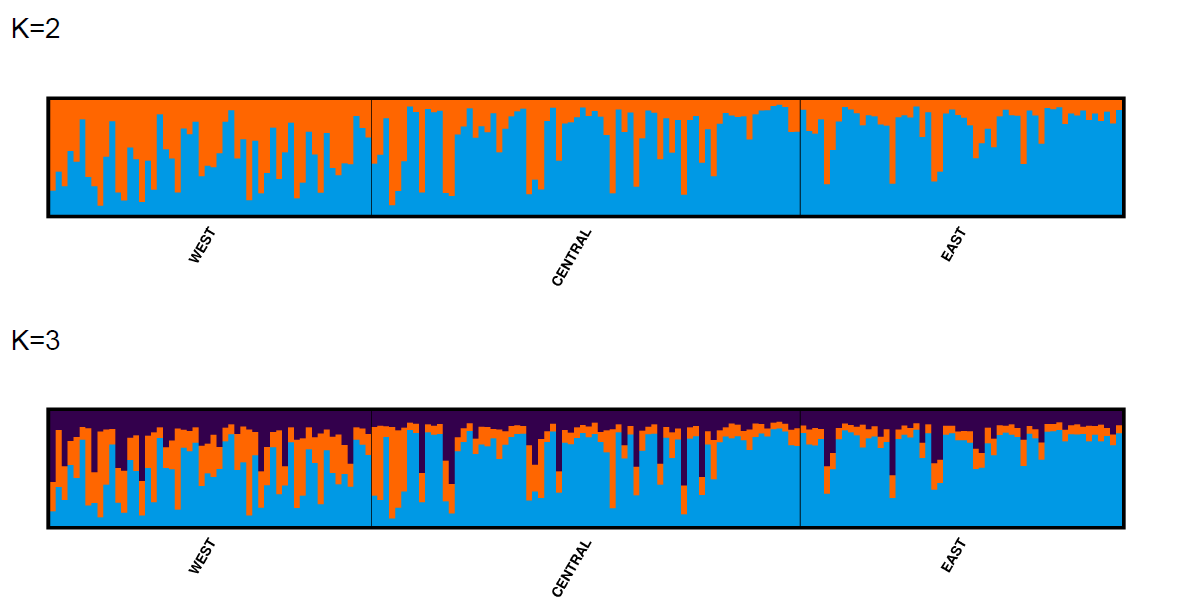


**Figure S4.** Principal Coordinate Analysis (PCoA) of all samples divided by sex (F = females, M = males). Samples from females found in the eastern distribution range and which received assignment scores q > 0.7 in the STRUCTURE analysis to a third genetic cluster (Fig. S2j), are highlighted in dark purple.

**Additional references:**

Puechmaille SJ. The program structure does not reliably recover the correct population structure when sampling is uneven: subsampling and new estimators alleviate the problem. Mol Ecol Resour. 2016;16(3): 608-627.
